# P24 Antimicrobial susceptibility profiles of hypervirulent strains of *Clostridioides difficile* of pig and human origin

**DOI:** 10.1093/jacamr/dlae136.028

**Published:** 2024-08-23

**Authors:** Aoife Doyle, Máire McElroy, Thomas Rogers

**Affiliations:** Clinical Microbiology, Trinity College Dublin, Dublin, Ireland; Department of Agriculture, Food and the Marine, Ireland; Department of Agriculture, Food and the Marine, Ireland; Clinical Microbiology, Trinity College Dublin, Dublin, Ireland

## Abstract

**Background:**

*Clostridioides difficile* places an increasing burden on healthcare because of emerging hypervirulent strains such as RT078/ST11 that may be resistant to commonly prescribed antibiotics e.g. fluoroquinolones. Pigs are often colonized by RT078/ST11 and act as potential reservoir for human *C. difficile* infection (CDI). They are the highest consumers of veterinary antimicrobials in many countries.^1^ We investigated the susceptibility of *C. difficile* to antibiotics that are commonly prescribed and/or used to treat CDI.

**Methods:**

Sequenced *C. difficile* (humans=50, pigs=40), collected as part of a One Health investigation into the epidemiology of pig and human *C. difficile*, were chosen for phenotypic antimicrobial susceptibility testing (AST). Strains included ST11 (*n*=70), ST44 (*n*=9), ST16 (*n*=6), ST8 (*n*=5). Susceptibility to a panel of antibiotics were determined using ANAERO3 Sensititre^™^ plates and Etest^®^, according to the manufacturer’s instructions. Selected *C. difficile* ST11 isolates (*n*=30) were tested for ciprofloxacin and delafloxacin susceptibility using Etest^®^. Breakpoints were interpreted as per EUCAST, CLSI and Freeman.^2^*C. difficile* genomes (*n*=90) were screened for AMR markers using NCBI AMRFinderPlus.

**Results:**

Phenotypic testing found all isolates were susceptible to vancomycin and metronidazole, and to penicillin, amoxicillin, amoxiclav, piperacillin/tazobactam, piperacillin, imipenem and chloramphenicol. Resistance was found to cefoxitin (100%), ceftriaxone (100%), erythromycin (48.94%), clindamycin (28.72%), moxifloxacin (48.94%), tetracycline (31.91%) and rifampicin (2.13%). All 30 ST11 isolates were resistant to ciprofloxacin, while the mean MIC for delafloxacin was 0.47 mg/L. There was no difference between resistance rate and *C. difficile* host. Bioinformatic analysis of 90 *C. difficile* genomes revealed resistance determinants for quinolones (87%) and tetracyclines (69%).

**Conclusions:**

No resistance to vancomycin and metronidazole in these *C. difficile* isolates is reassuring, although larger datasets for surveillance should be investigated. Finding resistance determinants in the isolates in this study demonstrates that WGS is a valuable tool for detecting AMR. Quinolone and tetracycline resistance may be linked with use of these antibiotics on Irish pig farms (64.1% and 85.1% of farms, respectively).^1^
